# Highly efficient differentiation of neural precursors from human embryonic stem cells and benefits of transplantation after ischemic stroke in mice

**DOI:** 10.1186/scrt292

**Published:** 2013-08-08

**Authors:** Danielle Drury-Stewart, Mingke Song, Osama Mohamad, Ying Guo, Xiaohuan Gu, Dongdong Chen, Ling Wei

**Affiliations:** 1 Department of Anesthesiology, Emory University, 101 Woodruff Circle, Atlanta, GA 30322, USA; 2 Department of Biomedical Engineering, Georgia Institute of Technology, 15 Ferst Drive, NW, Atlanta, GA 30332, USA; 3 Department of Biostatistics and Bioinformatics, Emory University, 1518 Clifton Road, NE, Atlanta, GA 30322, USA

**Keywords:** Human embryonic stem cell, Neural precursor, Electrophysiology, Stem cell, Cell therapy, Ischemic stroke, Neurogenesis, Small molecule

## Abstract

**Introduction:**

Ischemic stroke is a leading cause of death and disability, but treatment options are severely limited. Cell therapy offers an attractive strategy for regenerating lost tissues and enhancing the endogenous healing process. In this study, we investigated the use of human embryonic stem cell-derived neural precursors as a cell therapy in a murine stroke model.

**Methods:**

Neural precursors were derived from human embryonic stem cells by using a fully adherent SMAD inhibition protocol employing small molecules. The efficiency of neural induction and the ability of these cells to further differentiate into neurons were assessed by using immunocytochemistry. Whole-cell patch-clamp recording was used to demonstrate the electrophysiological activity of human embryonic stem cell-derived neurons. Neural precursors were transplanted into the core and penumbra regions of a focal ischemic stroke in the barrel cortex of mice. Animals received injections of bromodeoxyuridine to track regeneration. Neural differentiation of the transplanted cells and regenerative markers were measured by using immunohistochemistry. The adhesive removal test was used to determine functional improvement after stroke and intervention.

**Results:**

After 11 days of neural induction by using the small-molecule protocol, over 95% of human embryonic stem-derived cells expressed at least one neural marker. Further *in vitro* differentiation yielded cells that stained for mature neuronal markers and exhibited high-amplitude, repetitive action potentials in response to depolarization. Neuronal differentiation also occurred after transplantation into the ischemic cortex. A greater level of bromodeoxyuridine co-localization with neurons was observed in the penumbra region of animals receiving cell transplantation. Transplantation also improved sensory recovery in transplant animals over that in control animals.

**Conclusions:**

Human embryonic stem cell-derived neural precursors derived by using a highly efficient small-molecule SMAD inhibition protocol can differentiate into electrophysiologically functional neurons *in vitro*. These cells also differentiate into neurons *in vivo*, enhance regenerative activities, and improve sensory recovery after ischemic stroke.

## Introduction

Each year, approximately 795,000 people in the United States experience a stroke and it is now the fourth leading cause of death when considered separately from other cardiovascular diseases. It is also a leading cause of disability, and 26% of stroke survivors over 65 are still dependent on others for daily activities at 6 months after stroke [1]. However, administration of tissue plasminogen activator in the acute phase of stroke is still the only US Food and Drug Administration-approved treatment for this prevalent cause of death and morbidity and its application is limited by a narrow therapeutic window and a number of complications [2]. Other drugs that mediate significant neuroprotection in animal models and small trials, such as erythropoietin, have failed to demonstrate efficacy in large-scale human clinical trials [3].

Studies on treatment in the chronic phase of stroke are generally focused on recovering function through tissue repair and regeneration. Cell therapy is an attractive strategy for these goals, as transplanted cells may replace dead or damaged cells in addition to providing trophic support to supplement endogenous healing [4-6]. Various types of neural precursors, such as a conditionally immortalized cell line derived from human fetal tissue (now in clinical trials) [7-10], lines derived from carcinomas [11,12], fetal neuronal stem cells [13,14], mouse neural precursors derived from the post-stroke cortex [15], region-specific murine embryonic precursors [16], and precursors derived from mouse [17-19] or human [20-26] embryonic stem cells have been used in experimental models.

Human embryonic stem (hES) cells are pluripotent and can proliferate indefinitely in culture, both useful properties in the context of cell therapy. However, although neural differentiation is often seen as the default pathway for these cells [27], directed differentiation is difficult to optimize. Common neural differentiation protocols often use suspension culture techniques [28-33] or co-culture with feeder cells [31,34,35], both of which can introduce heterogeneous microenvironmental cues. Feeder cells, which are generally of rodent origin, also introduce xenogenic contaminants that can increase the immunogenicity of transplanted cells. Additionally, expensive recombinant factors like noggin are often used to obtain neural precursors [29,36-38], and the expense associated with these factors can be a limiting factor in scaling up cultures to the level required for preclinical development. More recently, there has been a greater focus on the use of fully adherent protocols [37] and small molecules [39] to cut down on heterogeneity and cost.

We previously reported the use of a fully adherent differentiation protocol that relies primarily on small molecules for differentiation, reducing the cost and heterogeneity in *in vitro* differentiation of neural precursors and neurons [40]. In the present study, we further characterize the *in vitro* differentiation of cells by using this protocol and demonstrate the use of hES cell-derived neural precursors in a murine model of ischemic stroke. We demonstrate that neural precursors derived by this method provide a useful cell population for cell-based stroke therapy.

## Methods

### Human embryonic stem cell maintenance and differentiation

H1 hES cells (p35-50; WiCell, Madison, WI, USA) were maintained on hES cell-qualified Matrigel (BD Biosciences, Sparks, MD, USA)-coated dishes in mTeSR1 medium (Stem Cell Technologies, Vancouver, BC, Canada). Differentiation was carried out as previously described [40]. Briefly, neural precursors were obtained by using a modified version of the differentiation protocol developed by Chambers and colleagues [37]. The neural precursors were seeded as single cells on growth factor reduced Matrigel (BD Biosciences)-coated dishes and grown to adherence, and SMAD inhibition was applied by using dorsomorphin (Tocris, Ellisville, MO, USA) and SB431542 (Stemgent, Cambridge, MA, USA). For *in vitro* differentiation of neurons, neural precursors were re-seeded as single cells and grown in a mixture of N2 and B27 medium (Invitrogen Corporation, Carlsbad, CA, USA) supplemented with 10 ng/mL basic fibroblast growth factor (bFGF) (R&D Systems, Minneapolis, MN, USA).

Differentiation was partially confirmed by staining by using standard protocols [41]. Cells were fixed in 4% paraformaldehyde (Sigma-Aldrich, St. Louis, MO, USA), permeabilized by using Triton-X-100 (G-Biosciences, St. Louis, MO, USA), blocked by using 1% fish gelatin (Sigma-Aldrich), and primary antibodies (nestin, neuronal nuclei (NeuN), neurofilament L (NF); Millipore, Billerica, MA, USA; paired box gene 6 (PAX6): Covance, Princeton, NJ, USA; sex-determining region Y-box 1 (SOX1): Santa Cruz Biotechnology, Santa Cruz, CA, USA) were applied overnight at 4°C in phosphate-buffered saline. Cy3- or Alexafluor 488-conjugated antibodies were applied for 1 to 2 hours at room temperature, and Hoechst 33324 (Invitrogen Corporation) or 4′,6-diamidino-2-phenylindole (DAPI) (Vector Labs, Burlingame, CA, USA) was used to counterstain nuclei. Cells expressing neural precursor markers were quantified by using the ImageJ cell counter, and at least 7,000 cells were counted per sample and no fewer than three samples were counted per marker.

Some antibodies were selected for Western blot analysis. Protein (30 μg) from each sample was loaded into a gradient gel and run at constant current until protein markers had adequately separated. They were transferred onto polyvinyl difluoride membranes that were then probed by using standard protocols. Primary antibodies (Actin, Sigma-Aldrich; glial fibrillary acidic protein (GFAP), Thermo Fisher Scientific, Waltham, MA, USA; GluA2, GluN3A, nestin, Millipore; GluN1, Cell Signaling, Danvers, MA, USA; Nav1.1, Abcam, Cambridge, MA, USA) were applied overnight at 4°C. Alkaline phosphatase (AP)- or horseradish peroxidase (HRP)-conjugated secondary antibodies were applied for 1 to 2 hours at room temperature. AP-conjugated antibodies were developed by using nitro-blue tetrazolium and 5-bromo-4-chloro-3'-indolyphosphate (NBT/BCIP) solution, and HRP-conjugated antibodies were developed by using a Pierce ECL Detection Kit (Thermo Fisher Scientific). Actin was used as a loading control.

### Electrophysiological recording of differentiating cells

Whole-cell patch clamp recording was performed on cultured cells exhibiting neuronal morphology at 7, 14, 21, and 28 days after re-seeding. The measurements were performed as in our previous studies by using an EPC9 amplifier (HEKA Elektronik, Lambrecht, Germany) at room temperature [42]. The external solution (pH = 7.4) contained 135 mM NaCl, 5 mM KCl, 2 mM MgCl_2_, 1 mM CaCl_2_, 10 mM HEPES, and 10 mM glucose. The internal solution (pH = 7.2) contained 120 mM KCl, 2 mM MgCl_2_, 1 mM CaCl_2_, 2 mM Na_2_ATP, 10 mM EGTA, and 10 mM HEPES. Recording electrodes were pulled from borosilicate glass pipettes (Sutter Instrument, Novato, CA, USA) and had a tip resistance of between 5 and 7 MΩ when filled with the internal solution. Series resistance was compensated by 75% to 85%. Linear leak and residual capacitance currents were subtracted online by using a P/6 protocol. Action potentials were triggered by depolarization pulses and recorded under current-clamp mode by using PULSE software (HEKA Elektronik), and data were filtered at 3 KHz and digitized at a sampling rate of 20 KHz.

Delayed rectifier potassium current densities (I_K_) were recorded from −60 to +60 mV with a 20 mV increment and a holding potential of −70 mV in the presence of 0.5 μM tetrodotoxin to block sodium currents. The reported current densities were measured at +40 mV. Transient outward potassium current densities (I_A_) were elicited from −60 to +40 mV with a 20-mV increment after a hyperpolarization of −110 mV for 500 ms. Peak amplitudes were measured at +40 mV. Both current measurements were normalized to cell size by using the capacitance of the cell.

### Ischemic stroke and cell transplantation

**Figure 1:**
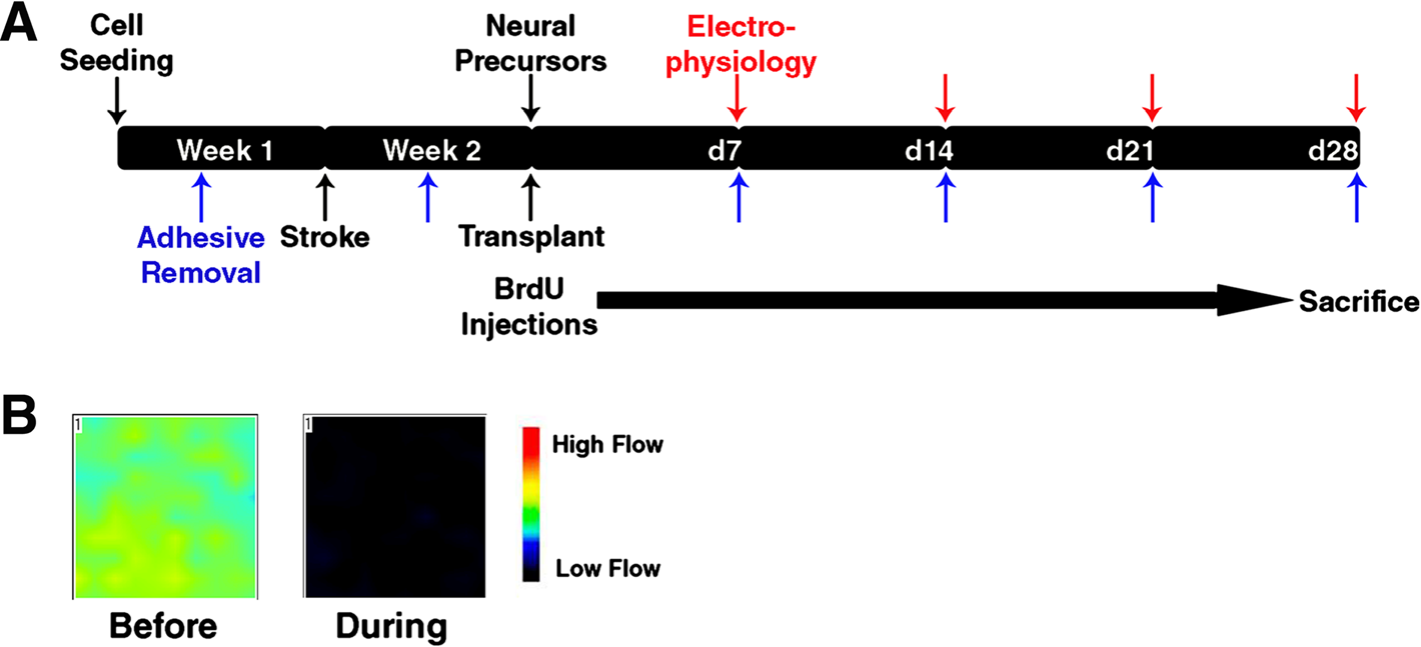
**Cell differentiation and stroke model. (A)** Timeline of the differentiation process, stroke induction, and testing. Neural precursors were obtained approximately 2 weeks after seeding, and electrophysiology measurements were carried out every 7 days after re-plating. Transplantation was carried out at the neural precursor stage, 1 week after stroke induction. Adhesive removal training took place in the week before stroke. Testing was carried out 4 days after stroke and then 7, 14, 21, and 28 days after transplantation. BrdU injections were given daily beginning on the day of transplantation. **(B)** Representative blood flow measurements before and during the 7-minute ligation of the CCA, demonstrating a nearly complete blood flow reduction to the affected area.

All procedures were approved by the Institutional Animal Care and Use Committee and met National Institutes of Health guidelines. Male 8- to 12-week-old C57/Bl6 mice were subjected to a focal ischemic stroke as previously described [41,43-45]. Briefly, during anesthesia with 2% chloral hydrate, a 2.0- to 2.5-mm-diameter craniectomy was performed through the right parietal skull, and the transparent dura was left intact over the whisker barrel cortex. Four to five distal branches of the middle cerebral artery serving the barrel cortex were ligated by using a 10-O suture through the dura. The creation of the whisker barrel ischemic region was completed by bilateral occlusion of the common carotid arteries (CCAs) for 7 minutes followed by reperfusion. Blood flow reduction was confirmed (Figure 1) by using the PeriScan PIM II Laser Doppler perfusion imager (Perimed AB, Cleveland, OH, USA) during the 7-minute ligation of the CCAs. This stroke model is focused on the whisker barrel cortex but also affects the forelimb region of the sensorimotor cortex.

Cell transplantation was carried out 7 days after stroke. Cells were labeled with a 1-hour exposure to 10 μg/mL Hoechst 33324 and treated with accutase to obtain a single-cell suspension. Cells were filtered through a 20-μm mesh and resuspended in N2 medium. Animals were re-anesthetized with 2% chloral hydrate. The ischemic infarction is well developed at this time, and the ischemic core (white or pale region) and peri-infarct area can be directly identified under an operating microscope through the cranial window. Injection of 4 μL of cell suspension (transplant group, 200,000 cells total) or medium (control group) into the core and penumbra regions of the stroke area was performed by using a Hamilton 80330 701 10-μL removable-needle syringe (Hamilton Company, Reno, NV, USA). Four injection sites (two in the core and two in the penumbra, 1 μL each) were used and each was carried out slowly (total injection time of 10 minutes). The needle was kept in the injection site for 2 minutes before withdrawal to prevent backflow of the injected solution. On the day of the transplant, animals began receiving daily intraperitoneal injections of 50 mg/kg bromodeoxyuridine (BrdU) (Sigma-Aldrich) to label proliferating cells. These injections were continued until sacrifice. Animals received no immunosuppression. Two to three days after injection, one transplant animal per group (n = 4) was sacrificed to assess graft survival. Staining was carried out on 10- to 20-μm sections by using the vendor instructions (DeadEnd™ Fluorometric TUNEL System; Promega Corporation, Madison, WI, USA).

### Immunohistochemistry

Animals were sacrificed 28 days after transplantation and brains were fresh-frozen in optimal cutting temperature compound (OCT) (Sakura Finetek, Torrance, CA, USA). Each 10-μm section on a slide was at least 100 μm from the previous section to avoid double-counting of cells, and slides were light-protected to preserve the Hoechst 33342 label. Slides were stained by using standard protocols for NeuN (Millipore) to label neurons, collagen IV (Col IV) (Millipore) to label vessels, or BrdU (AbD Serotec, Oxford, UK) to label newborn cells. Pictures were taken by using fluorescence microscopy along the length of the penumbra region defined morphologically as the region just outside the stroke core. Z-stack imaging was used to confirm co-localization. At least three sections per sample were quantified for each measurement. Where possible, human cells were identified by the Hoechst tag applied before transplantation.

### Hoechst-positive cell counting in brain sections

Cell count of Hoechst-positive cells remaining in the graft at 28 days was performed by following a modification of the principles of design-based stereology. Systematic random sampling was employed to ensure accurate and non-redundant cell counting. Every section under analysis was at least 100 μm away from the next. For each animal, six 10-μm-thick sections spanning the entire region of interest that crossed 600 μm around the ischemic core/peri-infarct region were counted. The total number of Hoechst-positive cells on the six sections of each slide was quantified.

### Behavioral testing

Starting several days before stroke, animals were trained in the adhesive removal task [46] until they could consistently completely remove the adhesive dot from both forepaws within 12 seconds. Both the time to contact and time to remove the dot after contact were recorded. Performance in this task was then measured 4 days after stroke to obtain a baseline measurement for impairment. Animals showing no impairment on the affected side were rare and were removed from further behavioral study. Testing was repeated 7, 14, 21, and 28 days after transplantation by an investigator blinded to the treatment groups.

### Statistical analysis

*In vitro* staining data are reported as the mean percentage ± standard deviation of cells positive for a marker. Peak voltage and current density values are reported as mean ± standard error of the mean (SEM) and were compared by using analysis of variance with a Tukey *post hoc* test. Peak voltage measurements of action potentials are pooled from cultures at 5% and 20% O_2_ tension. For *in vivo* immunohistochemical data, the values of mean ± SEM were compared by using a Student *t* test. Normal distribution was confirmed by using the Kolmogorov-Smirnov test.

A compound symmetry variance-covariance form in repeated measurements was assumed for each behavioral outcome. A signed rank test was used to determine the significance of post-stroke impairment. Post-stroke baseline measurements were compared between the control and transplant groups by using the Mann–Whitney *U* test. A repeated measures analysis using linear mixed models via SAS Proc Mixed (version 9.2; SAS Institute Inc., Cary, NC, USA) was performed for the longitudinal measurements of the time to contact and time to remove obtained at 7, 14, 21, and 28 days. The fixed effects in the models include the subject’s treatment group, time point, and interactions between treatment group and time point. The models also include the post-stroke baseline outcome measure (taken at 4 days) and the outcome measure of the unaffected forepaw at the same time point. These effects provide adjustment for different post-stroke performance levels across subjects and learning effects across time. The mixed linear models also include the subject-specific random intercept to account for between-subject random variability in outcome measures. All statistical tests were two-sided.

## Results

### Immunocytochemistry reveals highly efficient differentiation of neural precursors

**Figure 2:**
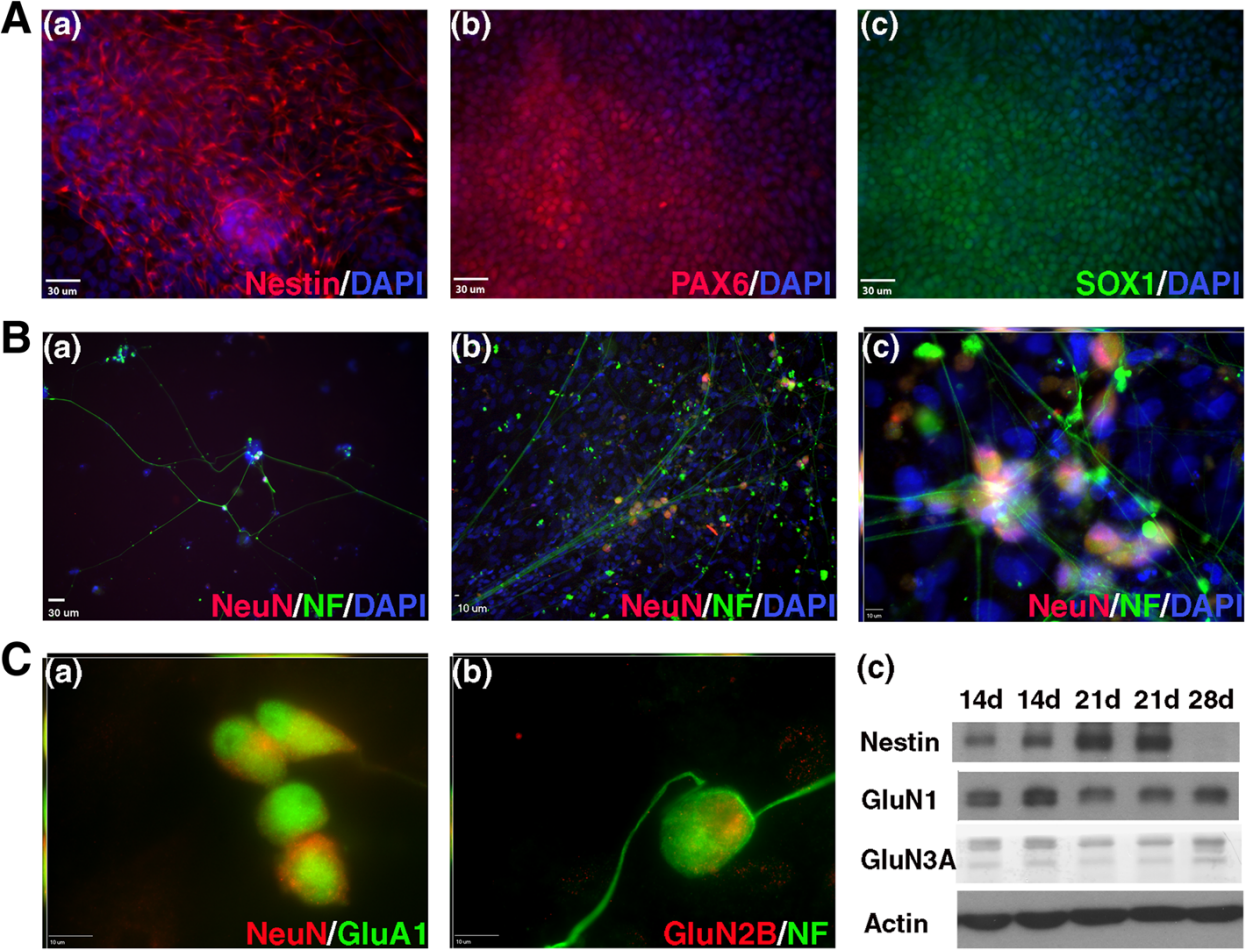
**hES cell-derived precursors express neural markers and differentiate into mature neurons *in vitro*. (A)** Neural precursors at day 11 after the beginning of SMAD inhibition; 96% ± 3% of cells expressed nestin (n = 4), 75% ± 7% expressed PAX6 (n = 4), and 64% ± 9% expressed SOX1 (n = 3). Scale bars are 30 μm. **(B)** After re-plating for terminal differentiation, cells positive for NeuN (red) and NF (green) were evident after 7 days (a) and persisted through 28 days (b). These cells formed large networks across the culture, and co-localization of NeuN and NF was evident (c). Scale bars are 10 μm. **(C)** At 28 days, cells expressed the AMPA and NMDA receptors GluA1, GluN1, GluN2B, and GluN3A. GluA1 and GluN2B are shown co-localized with NeuN and NF, respectively. Nestin was still present in these cultures through 21 days. Scale bars are 10 μm.

Neural differentiation of H1 hES cells was carried out by using a fully adherent SMAD inhibition protocol employing small molecules [37,40]. After 11 days of SMAD inhibition, cells had lost all detectable expression of pluripotency markers and had begun expressing neural precursor markers such as nestin, PAX6, and SOX1. Nestin is an intermediate filament protein that is expressed in the embryonic neuroepithelium and in neural precursors throughout the central nervous system [47,48]. PAX6 is an important transcription factor in cortical development [49,50] and is necessary for the development of the thalamocortical tract [51]. We previously reported the expressions of these markers after SMAD inhibition with SB431542 and dorsomorphin as 96% ± 3% and 75% ± 7%, respectively [40]. In the present study, we examined the expression of SOX1, another transcription factor indicated in the specification of early neural cell fate [52]. This marker was expressed in 64% ± 9% of cells after 11-day differentiation (Figure 2A). Taken together, these markers indicate efficient differentiation into neural precursors, and most of the cells are biased toward a forebrain lineage.

Staining was also used to verify the ability of these neural precursor cells to differentiate into neurons *in vitro*. In a mixture of N2 and B27 media, cells formed well-connected networks expressing NeuN and NF (Figure 2B). These cells also expressed β-III tubulin and microtubule-associated protein 2 (MAP2) (data not shown). The neuronal markers were evident as early as 7 days after re-plating for terminal differentiation (Figure 2B-a) and persisted through 4 weeks of culture (Figure 2B-b,c). In addition to these general markers, cells with a neuronal morphology expressed the 2-amino-3-(3-hydroxy-5-methyl-isoxazol-4-yl)propanoic acid (AMPA) receptor subunit GluA1 and the N-methyl-D-aspartic acid (NMDA) receptor subunit GluN2B (Figure 2C). Western blotting also revealed the presence of the NMDA receptor subunits GluN1 and GluN3A (Figure 2C-c), the AMPA receptor subunit GluA2, and the sodium channel subunit (Nav1.1) (data not shown). Nestin expression was still present in the cultures at days 14 and 21, suggesting that some of the underlying cells were still precursors. However, this expression was lost by day 28 (Figure 2C-c). GFAP was also detected by Western blotting at 14, 21, and 28 days of terminal differentiation, suggesting astrocytic differentiation (not shown).

### Human embryonic stem cell-derived neuronal cells display functional electrophysiological properties *in vitro*

**Figure 3:**
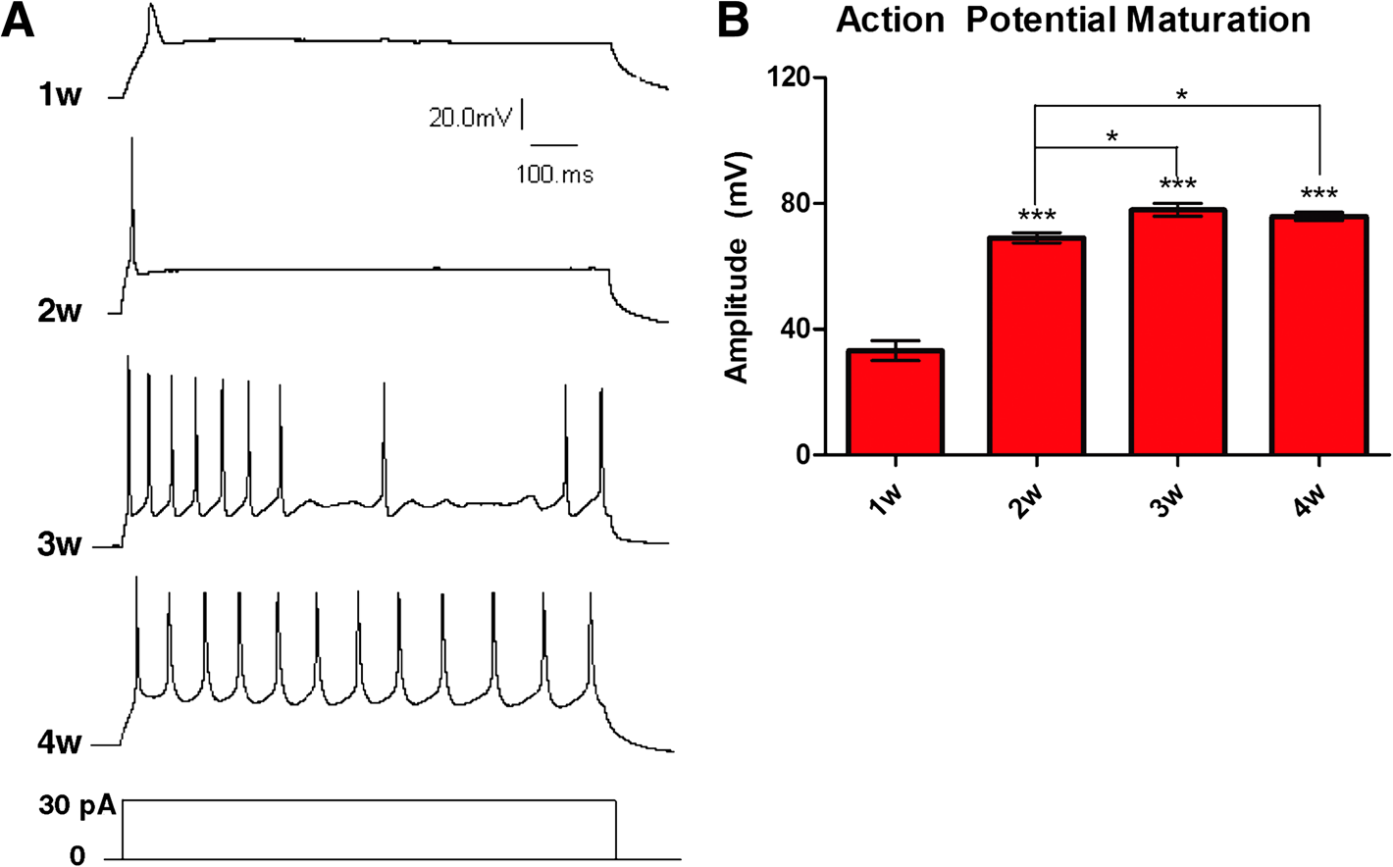
**hES cell-derived neurons exhibit mature action potential responses. (A)** Representative traces of action potentials fired in response to depolarization at 1, 2, 3, and 4 weeks after re-plating for terminal differentiation. Approximately 1 out of 7 and 1 out of 3 cells fired repetitive trains of action potentials at 3 and 4 weeks, respectively. **(B)** Quantification of the maximum action potential amplitude presented as mean ± SEM. A significant increase was observed from 1 to 2 weeks and again from 2 to 3 weeks. n = 19 (1w), 23 (2w), 26 (3w), and 47 (4w). *P <0.05, ***P <0.001.

To measure electrophysiological function in hES cell-derived neuronal cells, we performed whole-cell patch-clamp recording over the course of 4 weeks of differentiation. Action potentials displayed a pattern of maturation over the 4-week differentiation period (Figure 3). At 1 week, the evoked response was slow and weak, and the mean amplitude was 33.2 ± 3.2 mV. After 2 weeks of terminal differentiation, most cells fired significantly stronger action potentials with single sharp spikes at a mean amplitude of 69.1 ± 1.7 mV. Further maturation increased this response to a mean amplitude of 78.0 ± 2.0 mV at 3 weeks, and there was no further significant change at 4 weeks. Three weeks of terminal differentiation was also the point at which repetitive trains of action potentials were first observed, and approximately 1 out of 7 of cells exhibited multiple action potentials in response to a single depolarization event. Although no significant change in amplitude was observed from 3 to 4 weeks of differentiation, the proportion of cells firing repetitive trains increased to approximately 1 out of 3 of the cells examined. Miniature excitatory post-synaptic potentials were evident in cultures at all measured time points (data not shown), indicating functional synapse formation between cells.

**Figure 4:**
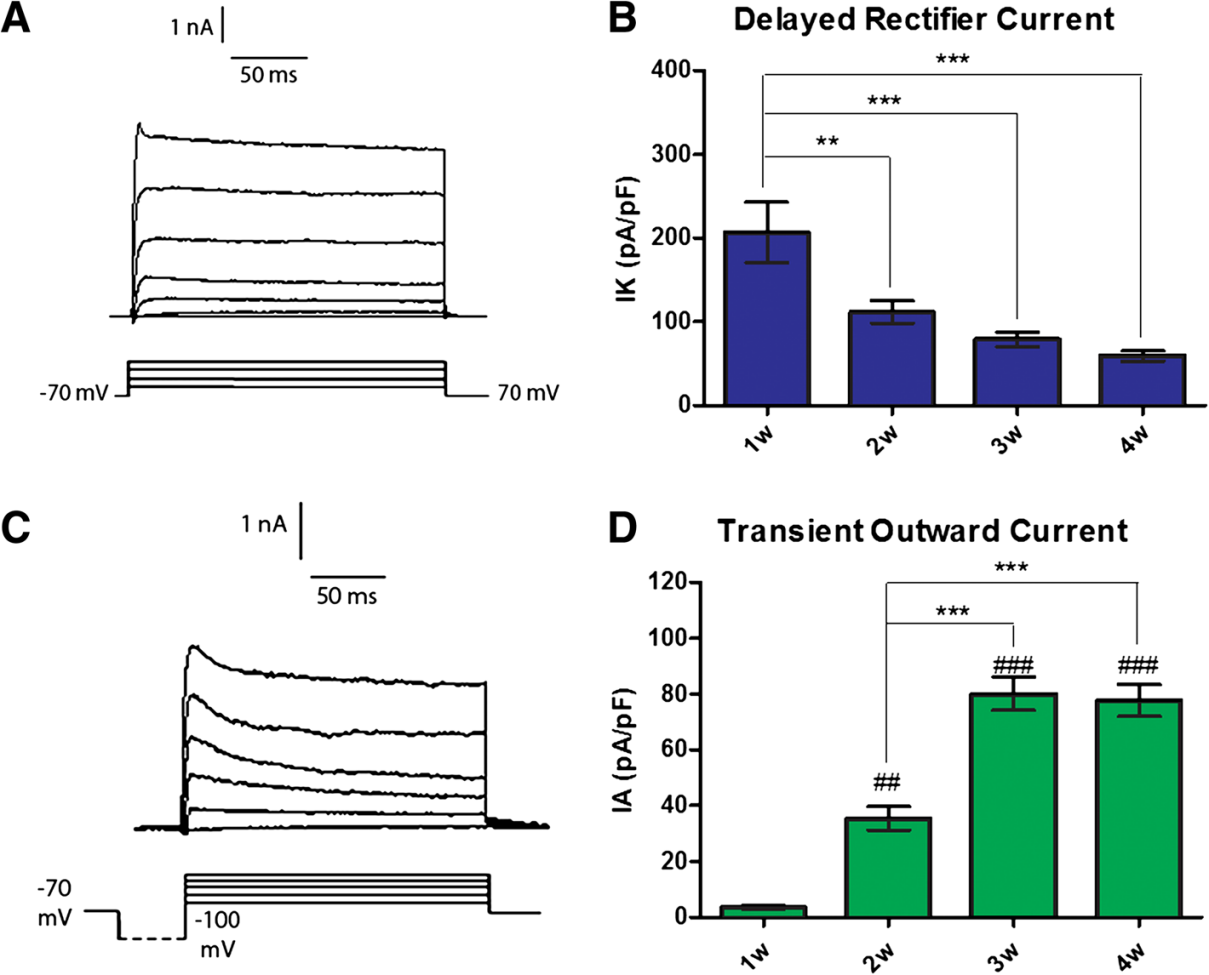
**Potassium currents in hES cell-derived neurons mature over 4 weeks in culture. (A)** Representative traces of the delayed rectifier K^+^ current in hES cell-derived neurons. **(B)** Quantification of the peak delayed rectifier current density at +40 mV. The current densities measured in the first week of terminal differentiation were significantly higher than all later measurements. **(C)** Representative traces of the transient outward K^+^ current in hES cell-derived neurons. **(D)** Quantification of the peak transient outward current density at +40 mV. Current densities significantly increased from week 1 to week 2 and then again at week 3. n = 7 (1w), 9 (2w), 11 (3w), 12 (4w). **P <0.01, ***P <0.001, ^##^*P* <0.01 as compared with 1 week, ^###^*P* <0.001 as compared with 1 week.

We also examined changes in potassium currents in differentiating cells (Figure 4). The delayed outward rectifier current density declined over time with neuronal differentiation, from 206.6 ± 36.4 pA/pF at 1 week to 111.2 ± 13.2 pA/pF at 2 weeks. This decreasing trend continued over time, but no further statistically significant change was observed between weeks 3 and 4 of terminal differentiation. The fast transient outward current density, on the other hand, increased over time. Very small current densities of 3.6 ± 0.5 pA/pF were observed at 1 week, increasing to 35.4 ± 4.3 pA/pF at 2 weeks and 80.2 ± 6.0 pA/pF at 3 weeks into terminal differentiation. Again, no further change was noted at 4 weeks of differentiation. It is likely that the increased role of the transient outward K^+^ current contributed to the maturation of the action potential response by allowing cells to repolarize more quickly.

### Human embryonic stem cell-derived neural precursors survive and differentiate into neurons *in vivo*

**Figure 5:**
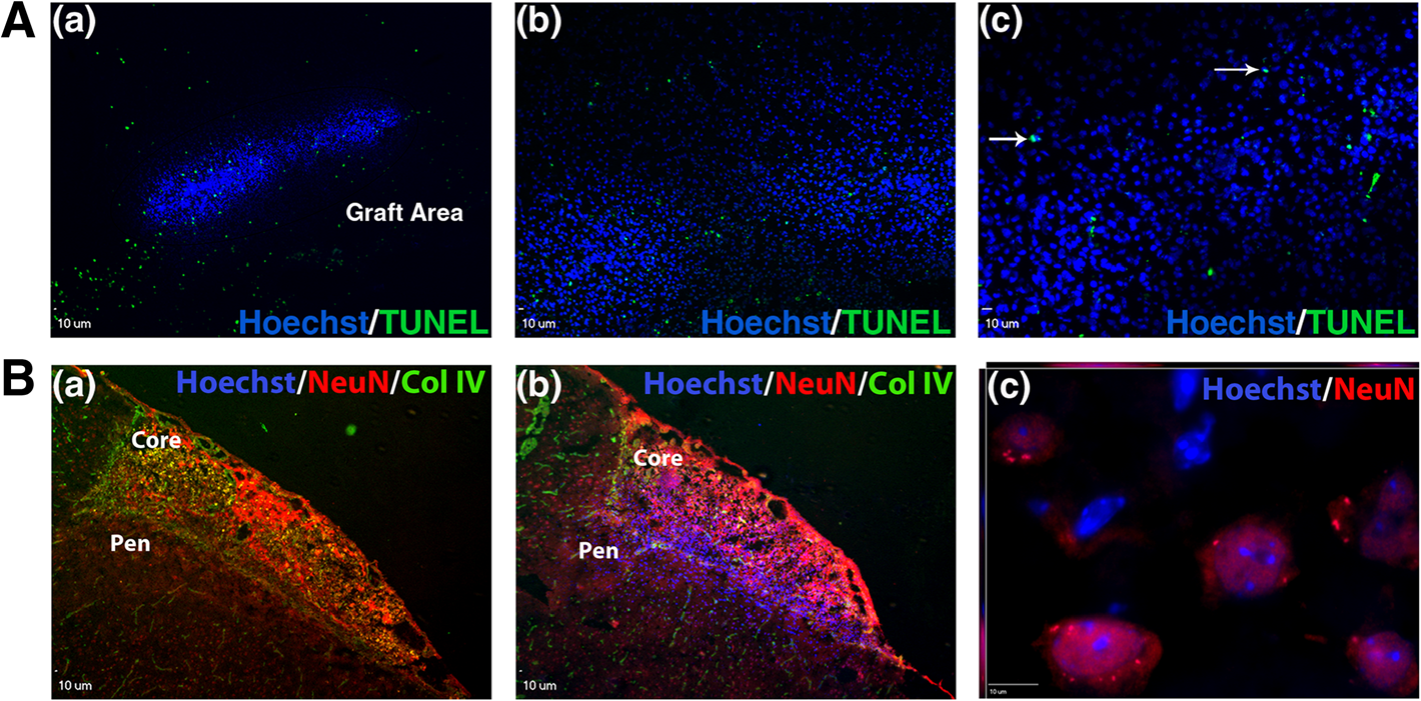
**hES cell-derived neural precursors survive and differentiate into neurons *in vivo*. (A)** Representative example of graft survival 2 days after transplantation. The circle in (a) marks the graft area as determined by the Hoechst-tagged precursor cells. Arrows in (c) demonstrate occasional TUNEL co-localization with Hoechst-positive cells. **(B)** Representative images stained for NeuN (red) and collagen IV (green) 28 days after transplantation. No Hoechst-positive cells (blue) are evident in the medium control animal (a), whereas many transplanted cells are still present in the transplant animal (b). The high-power image in (c) demonstrates co-localization of Hoechst 33342 and NeuN in the penumbra region, indicating neuronal differentiation of transplanted cells. All scale bars = 10 μm.

hES cell-derived neural progenitors were transplanted into the ischemic core and penumbra regions 7 days after ischemic stroke. Two to three days after transplantation, one animal per group (n = 4) was sacrificed to check for cell survival. Transplanted cells were identified by using the Hoechst tag, and TUNEL staining was used to assess cell death. Although there was some TUNEL staining present in the graft area, co-localization with Hoechst-positive cells was negligible (Figure 5A). Cell survival was further indicated by the fact that the Hoechst tag was still visible in a large number of cells in the stroke core and penumbra of transplant animals after 28 days *in vivo* (Figure 5B-b). Hoechst 33342-positive cells in the grafted core/peri-infarct region were 5,541 ± 180 (averaged total number from six sections per animals; n = 3 mice). Neuronal differentiation of transplanted cells was assessed by quantifying Hoechst 33342 co-localization with NeuN in the penumbra region (Figure 5B-c). At day 28, the percentage of Hoechst-positive cells that were also NeuN-positive was highly variable between animals, ranging from 13.5% to 40.8%, and the mean value was 20.6% ± 10.3%. Hoechst 33342 co-localization with MAP2 was also observed (data not shown), further indicating neuronal differentiation. Some Hoechst-positive cells (14.6% ± 2.4%) did co-localize with vessels, but whether the cells were part of the vessels or migrating along them was not clear.

### Transplantation increases the proportion of bromodeoxyuridine-positive neurons at 28 days

**Figure 6:**
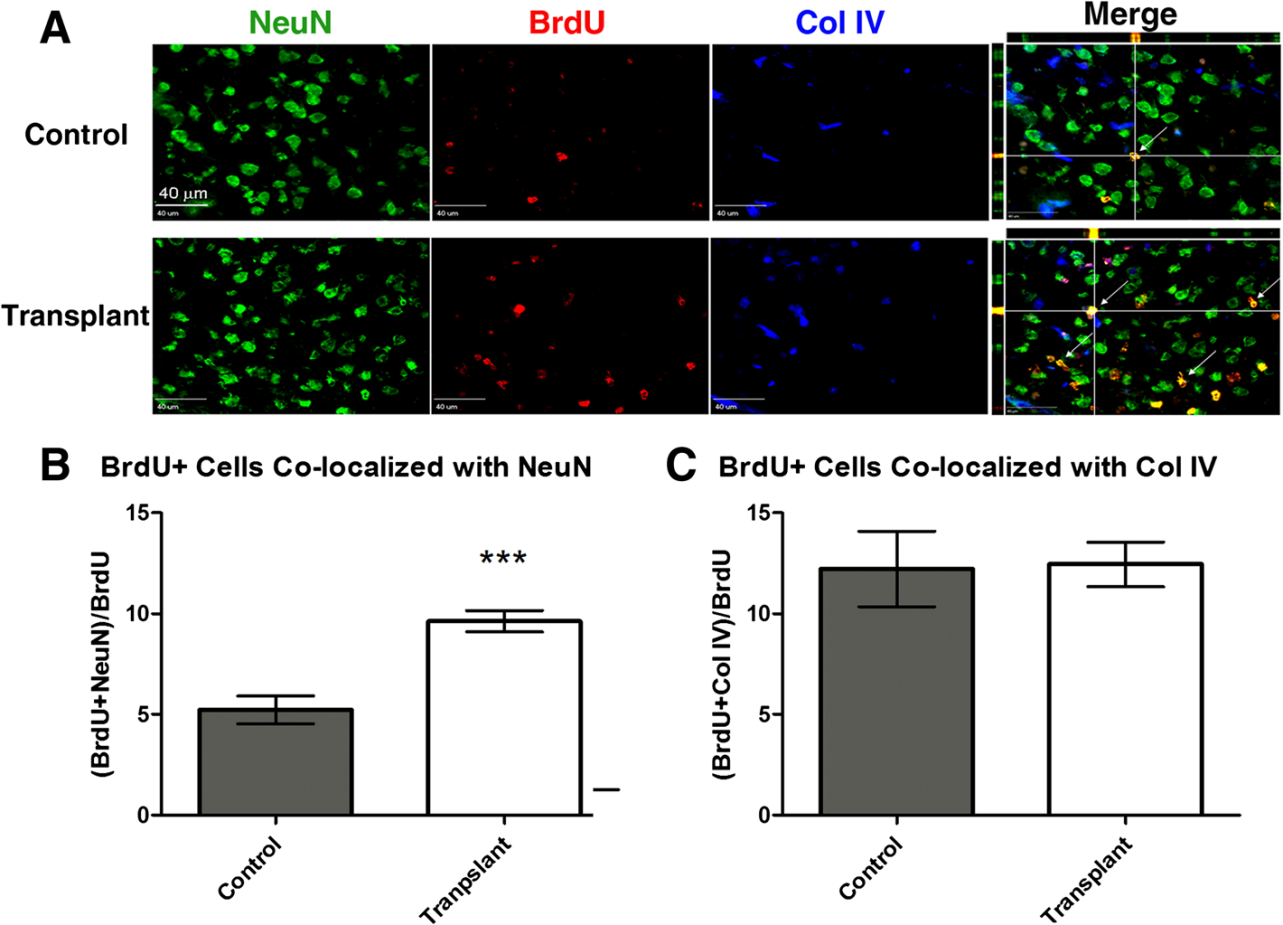
**Transplantation of hES cell-derived neural precursors increases BrdU co-localization with NeuN. (A)** Representative images of staining for NeuN (green), BrdU (red), and Col IV (blue) in the penumbra region of a medium control and a cell transplant animal. White arrows indicate co-localization of NeuN and BrdU. Scale bars = 40 μm. **(B)** A significantly higher percentage of BrdU-positive cells in the transplant animals co-localized with NeuN as compared with control animals, potentially indicating a higher degree of neurogenesis. ***P <0.001, n = 6 (control) and 8 (transplant). **(C)** No significant difference was observed in the percentage of BrdU-positive cells co-localized with Col IV, indicating no difference in angiogenesis. n = 6 (control) and 7 (transplant).

Animals received daily injections of BrdU beginning on the day of the transplant in order to track the fate of newborn cells. No difference in the total number of BrdU-positive cells in the penumbra was observed between animals receiving cell transplantation and medium controls. However, the percentage of BrdU-positive cells co-localized with NeuN was 9.6% ± 0.5% in the transplant group (n = 8), which is significantly higher than the 5.2% ± 0.7% observed in the control group (n = 6) (Figure 6). This indicates an increase in the number of newborn neurons in the transplant group, although BrdU may also be incorporated into cells undergoing DNA repair. The fact that the total density of BrdU-positive cells was not increased in the transplant group suggests that proliferation in the graft was low. This supports the hypothesis that BrdU incorporation represents endogenous regenerative activity but does not fully exclude the contribution of proliferation or repair in graft-derived cells.

The number of BrdU-positive cells co-localized with Col IV was examined as a marker of angiogenesis. In this case, no significant increase was observed; 12.2% ± 1.9% of BrdU-positive cells co-localized with Col IV in the control group (n = 6) and 12.4% ± 1.1% in the transplant group (n = 7) (*P* >0.05; Figure 6).

### Transplantation improves functional recovery after stroke

Functional recovery was assessed by using the adhesive removal test. On the affected side, both the time to contact and the time to remove were significantly longer after stroke (Table 1). As expected, neither of these measures was significantly different in either group on the unaffected side. The post-stroke baseline measurements were not significantly different between the control and transplant groups (Table 2). The model-based estimate of the mean outcome measures at each time point was analyzed (Table 3). It is important to note that these are not absolute measurements but instead are estimates that incorporate the post-stroke impairment and the learning curve observed on the unaffected side. There was no significant difference between the outcome measures between the control and transplant groups at any of the four time points. However, the estimated slope over time in the mixed linear model of the longitudinal outcome measures reveals a significant decreasing trend in time to contact in the transplant group (estimated slope is −0.15, *P* value = 0.012). This trend is not observed in the control group (estimated slope is 0.06, *P* value = 0.314). The time to remove does not change significantly across time in either group. This suggests an improvement in the sensory function of the forepaw in transplant animals as compared with controls. Additionally, the variance of the control group was significantly higher than that of the transplant group in both measures, indicating a more consistent recovery in treated animals.

**Table 1:** Performance in the adhesive removal task is significantly altered by stroke

**Measurements**		**Control group**	**Transplant group**
		**(n = 17)**	**(n = 18)**
Affected side	Time to contact	*P* = 0.001	*P* = 0.001
	Time to remove	*P* <0.0001	*P* = 0.0004
Unaffected side	Time to contact	*P* = 0.627	*P* = 0.255
	Time to remove	*P* = 0.220	*P* = 0.091

**Table 2:** No difference was found between groups in post-stroke performance

**Measurements**		**Control group**	**Transplant group**	***P *** **value**
		**(n = 17)**	**(n = 18)**	
Affected side	Time to contact	10.78 ± 15.17	9.26 ± 10.33	0.644
	Time to remove	11.24 ± 8.02	20.25 ± 32.48	0.381
Unaffected side	Time to contact	3.16 ± 3.90	2.65 ± 2.96	0.842
	Time to remove	5.97 ± 2.40	7.33 ± 4.13	0.363

**Table 3:** Time to contact improves over time in animals receiving transplant

**Measurements**		**Estimated mean (SE)**				**Slope across time**	
		**7 days**	**14 days**	**21 days**	**28 days**	**Estimated slope (SE)**	***P *** **value**
Time to contact	Transplant	7.19 (1.20)	5.06 (1.19)	4.65 (1.19)	3.75 (1.18)	−0.15 (0.06)	0.012
	Control	5.87 (1.26)	4.21 (1.22)	4.79 (1.22)	4.11 (1.22)	−0.06 (0.06)	0.314
	*P* value	0.442	0.617	0.936	0.833		
Time to remove	Transplant	10.74 (0.95)	9.30 (0.95)	9.55 (0.95)	10.10 (0.95)	−0.02 (0.05)	0.652
	Control	9.95 (0.97)	7.99 (0.96)	9.55 (0.97)	10.21 (0.99)	0.04 (0.05)	0.497
	*P* value	0.567	0.351	0.998	0.936		

*SE* standard error.

## Discussion

This study details the use of a fully adherent and feeder-free differentiation protocol employing small molecules to obtain neural precursors for transplantation after stroke. Although there is still some batch-to-batch variation and some animal products are still used, this protocol reduces the heterogeneity present in suspension culture and reduces possible contamination from animal products by removing the use of feeder cells in cell culture. Here, we demonstrate that neural precursors derived by using this protocol can develop into electrophysiologically active neurons, suggesting that they have the potential to act as functional neurons in damaged tissue. We further demonstrate that neural precursors survive, differentiate into neurons, improve neural regeneration, and enhance sensory function after transplantation into the penumbra region of stroke.

As we demonstrated during terminal differentiation, it is possible for cells to express neuronal markers like NeuN and neurofilament without exhibiting mature electrophysiological function. It is important to ensure that cells intended to replace lost tissue in the brain can further differentiate into neurons and that those neurons can respond appropriately to electrical signals. However, many studies rely on protein expression, without testing for electrophysiological function. Johnson *et al*. [28] studied functional development in hES cell-derived (H9) neurons over the course of 7 weeks of terminal differentiation (10 weeks from the onset of differentiation in hES cells). PAX6^+^/SOX1^+^ progenitors were obtained within 2 weeks by using suspension culture and neural rosette isolation, similar to our time course. These were again cultured in suspension for 1 week before plating for terminal differentiation in a medium containing brain-derived neurotrophic factor and glial cell line-derived neurotrophic factor, among other factors. Electrophysiological properties were examined at 1, 3, 4, and 7 weeks after plating for terminal differentiation. High-amplitude, single-spike action potentials were first reported at 4 weeks of terminal differentiation, and repetitive trains were observed in some cells at 7 weeks. In contrast, we obtained high-amplitude, single-spike action potentials at only 2 weeks after plating for terminal differentiation, and bFGF was the only growth factor added to our base medium. We began to obtain repetitive trains at 3 weeks of terminal differentiation, and the proportion of cells firing them increased with another week of culture. We have therefore greatly reduced the time and cost associated with obtaining electrophysiologically active neurons *in vitro*. After transplantation, cells differentiated into neuronal cells. Although the present study could not verify the electrophysiological properties of these cells, behavioral tests support the hypothesis that the transplanted cells participated in functional repair of damaged brain structures.

In this study, we examined neuronal differentiation *in vitro* to confirm the ability of our hES cell-derived neural precursors to further differentiate into neurons. While these neurons were expressing receptor subunits and electrical activity consistent with an excitatory phenotype, we did not determine the exact subtype of neurons we derived *in vitro*. This determination, along with how environments approximating the stroke and penumbra region affect the differentiation, will be important as therapies move toward the clinic. To this same end, it will be important to further identify the non-neuronal cells in culture with an emphasis on demonstrating that the cell types derived become post-mitotic upon differentiation and do not form any inappropriate cell types. In this study, we used bFGF as our only recombinant growth factor, but it is possible that further patterning factors will increase the proportion of neurons in culture and permit the derivation of specific subtypes. Patterning factors are usually recombinant growth factors that can greatly increase the cost of culture, but small molecules may lead to decreased cost in this arena as well. For example, purmorphamine is a sonic hedgehog (shh) agonist that has been used in the derivation of dopaminergic neurons [53].

*In vivo*, we observed a very high degree of cell survival after transplantation. This may have been due, in part, to the presence of Matrigel throughout the differentiation process. It was recently reported that hES cell-derived neural precursors cultured with Matrigel before transplantation or injected with a Matrigel scaffold reduced infarct size, improved behavioral outcomes, and differentiated primarily into neuronal cells [22]. However, cells that were not exposed to Matrigel exhibited high levels of cell death and lower proportions of neuronal markers and did not improve infarct size or behavioral outcomes. We have also reported positive effects of Matrigel on hES cell-derived neural precursors *in vitro*, where we found that cells terminally differentiated on poly-D-lysine/laminin-coated dishes never developed mature action potential responses but that those grown on Matrigel-coated dishes did [40]. Thus, the use of Matrigel throughout our differentiation process may have contributed to the positive results we report here. However, Matrigel will need to be removed from the process if it is ever used in human trials, as the removal of xenogenic products is largely seen as necessary for widespread clinical use [54].

One major concern with the use of pluripotent stem cells in cell therapy is the fear of tumor formation. We did not observe any teratoma formation and this was likely due the lack of residual pluripotent cells in our cultures. However, pluripotent cells need not be present for tumor formation. For example, small rosette-like tumors can form if hES cell-derived neural precursors are transplanted at a stage of differentiation in which cells are highly proliferative but not yet similar to fetal brain in the expression of neural markers [24]. These cells expressed high levels of PAX6 but had only low-level expression of SOX1, whereas the neural precursor stage, which did not result in any tumor formation, expressed high levels of both markers. The cells we obtain with small-molecule SMAD inhibition also highly expressed both of these markers, and we observed no adverse effects from cell proliferation in the brain tissue. In fact, the total numbers of BrdU-positive cells found in the control and transplant groups were not significantly different, suggesting low levels of proliferation in the graft.

Another concern in our model may have been the use of the Hoechst tag for tracking, as it has the potential to cause problems in DNA replication or leak into neighboring cells. However, this tag has been successfully used in prior studies [17], and we did not observe any tumor formation *in vivo*. As argued earlier, it is likely that the transplanted cells were not proliferative, mitigating any problems with DNA replication. In future studies, a better tracking method may be something akin to that used by Daadi *et al*. [25]. Transfection of cells with an easily identifiable marker that does not leak and can be easily co-stained would be ideal. The additional inclusion of a bioluminescent marker or superparamagnetic iron oxide (SPIO) tag would also allow for *in vivo* monitoring in live animals.

A different tracking method would also help to better differentiate between endogenous and graft-derived neurogenesis. In previous studies with this stroke model, we have shown that newborn cells that express doublecortin (DCX) and incorporate BrdU migrate from the SVZ to the stroke region and form new neurons. Other interventions, such as whisker stimulation, can increase the number of neuroblasts migrating toward the stroke region at early time points and new neurons in the penumbra region 4 weeks after stroke [55]. This response could be enhanced by the transplantation of neural precursors, but this hypothesis remains to be verified by using more specific markers and technologies.

BrdU incorporation is the most commonly used and clearest measure for tracking the fate of newborn cells in the nervous system [56,57], as it remains in the cell even after it differentiates and leaves the cell cycle, but there are concerns with its use. Because BrdU incorporates during DNA replication, it is possible that labeled cells may have been undergoing cell repair (successfully or prior to apoptosis) rather than mitosis [58]. In unpublished studies, we have found little to no co-staining of TUNEL and BrdU in this model, and none of the counted cells showed morphological signs of apoptosis or necrosis, so it is unlikely that the BrdU-positive cells we observed were dying. It is also unlikely that our dose of 50 mg/kg would be sufficient to visualize cells undergoing repair [59], although an increase in successful cell repair would also be a desirable outcome of transplantation. Future studies will need to differentiate between cell repair and true neurogenesis by examining earlier time points and quantifying DCX-positive neuroblasts migrating to the stroke region after treatment.

Our previous unpublished studies have demonstrated no change in graft survival in this model when immune suppression is administered and we chose not to use it in this study. However, it is possible that we would have been able to achieve greater or more consistent levels of neuronal differentiation *in vivo* if immune suppression had been used. In a transplant model using mouse embryonic stem-cell derived neurospheres, graft survival was unchanged by the administration of cyclosporine A, but inflammatory factors biased cells toward glial differentiation rather than neuronal differentiation when it was not given [60]. This may have occurred in our model as well, although we likely mitigated this effect by transplanting 7 days after stroke, when inflammation in the stroke region has largely subsided. It is also important to note that immune suppression may be detrimental to healing after stroke. Inflammatory signals can attract stem cells to the site of injury [61], and the immune response may be neuroprotective and necessary for endogenous neurogenesis [62-65]. Additionally, the immune system is already naturally suppressed after stroke [66], and further suppression may increase the risks of infection and tumor formation [67,68]. It is thus clear that systemic immune suppression in patients with stroke should be avoided wherever possible.

## Conclusions

We have demonstrated that neural precursors derived from hES cells by using small-molecule SMAD inhibition in a fully adherent protocol can differentiate into neurons both *in vitro* and *in vivo* after transplantation into the ischemic brain. This protocol reduces the heterogeneity, cost, and use of animal products in obtaining hES cell-derived neural precursors that can differentiate into electrophysiologically active neurons and allows for transplantation at a safe stage of differentiation. Transplantation of these cells improves regenerative activities and sensory function even without immune suppression. Further studies will be needed to fully characterize the integration of these cells into the damaged tissue and the paracrine effects on endogenous healing.

## Abbreviations

AMPA: 2-amino-3-(3-hydroxy-5-methyl-isoxazol-4-yl)propanoic acid; AP: Alkaline phosphatase; bFGF: Basic fibroblast growth factor; BrdU: Bromodeoxyuridine; CCA: Common carotid arteries; Col IV: Collagen IV; DCX: Doublecortin; GFAP: Glial fibrillary acidic protein; hES: Human embryonic stem; HRP: Horseradish peroxidase; MAP2: Microtubule-associated protein 2; NeuN: Neuronal nuclei; NF: Neurofilament L; NMDA: N-methyl-D-aspartic acid; PAX6: Paired box gene 6; SEM: Standard error of the mean; SOX1: Sex-determining region Y-box 1; SVZ: Subventricular zone; TUNEL: Terminal deoxynucleotidyl transferase dUTP nick end labeling.

## Competing interests

The authors declare that they have no competing interests.

## Authors’ contributions

DD-S carried out the cell and tissue processing, staining, Western blots, and most statistical analyses; participated in the study design; and drafted the manuscript. MS carried out the electrophysiological measurements. OM aided in cell culture, study design, and manuscript revision. YG designed and carried out the statistical analysis of the adhesive removal tests and contributed to the drafting of the manuscript. XG carried out all animal surgeries. DC aided in immunohistochemistry and cell quantification. LW helped in initiation of the research idea, participated in the study design, and revised the manuscript. All authors read and approved the final manuscript.
